# 2,6-Diamino­pyridinium bis­(4-hydroxy­pyridine-2,6-dicarboxyl­ato-κ^3^ *O* ^2^,*N*,*O* ^6^)chromate(III) dihydrate

**DOI:** 10.1107/S1600536808027347

**Published:** 2008-08-30

**Authors:** Hossein Aghabozorg, Leila Roshan, Najmeh Firoozi, Mohammad Ghadermazi, Sara Bagheri

**Affiliations:** a Faculty of Chemistry, Tarbiat Moallem University, 49 Mofateh Avenue 15614, Tehran, Iran; b Department of Chemistry, Faculty of Science, University of Kurdistan, Sanandaj, Iran; c Faculty of Chemistry, Islamic Azad University, North Tehran Branch, Tehran, Iran

## Abstract

The reaction of chromium(III) nitrate hexa­hydrate, pyridine-2,6-diamine and 4-hydroxy­pyridine-2,6-dicarboxylic acid in a 1:2:2 molar ratio in aqueous solution resulted in the formation of the title compound, (C_5_H_8_N_3_)[Cr(C_7_H_3_NO_5_)_2_]·2H_2_O or (pydaH)[Cr(hypydc)_2_]·2H_2_O (where pyda is pyridine-2,6-diamine and hypydcH_2_ is 4-hydroxy­pyridine-2,6-dicarboxylic acid). Each Cr^III ^atom is hexa­coordinated by four O and two N atoms from two (hypydc)^2−^ fragments, which act as tridentate ligands, in a distorted octa­hedral geometry. The O—Cr—O—C torsion angles between the two planes of the (hypydc)^2−^ fragments [−99.81 (17) and 97.77 (17)°] indicate that these two units are almost perpendicular to one another. In the crystal structure, extensive O—H⋯O, N—H⋯O and C—H⋯O hydrogen bonds with *D*⋯*A* distances ranging from 2.560 (2) to 3.279 (3) Å, ion pairing, C—O⋯π [O⋯π = 3.166 (2) Å] and π–π stacking inter­actions between (hypydc)^2−^ and (pydaH)^+^ rings [with a centroid–centroid distance of 3.3353 (14) Å] contribute to the formation of a three-dimensional supra­molecular structure.

## Related literature

For related literature, see: Aghabozorg *et al.*, (2007 ▶); Aghabozorg, Manteghi *et al.* (2008 ▶); Aghabozorg, Saadaty *et al.* (2008 ▶); Ranjbar *et al.* (2001 ▶); Soleimannejad *et al.* (2008 ▶).
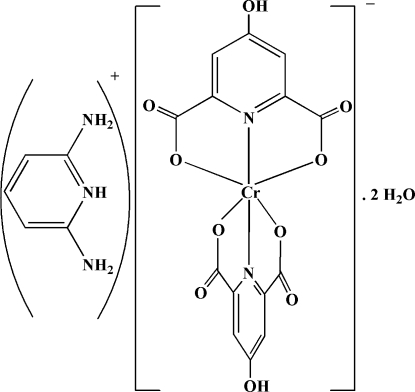


## Experimental

### 

#### Crystal data

(C_5_H_8_N_3_)[Cr(C_7_H_3_NO_5_)_2_]·2H_2_O
*M* *_r_* = 560.38
Monoclinic, 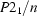

*a* = 6.9590 (4) Å
*b* = 20.9904 (12) Å
*c* = 14.8951 (8) Å
β = 95.3030 (13)°
*V* = 2166.4 (2) Å^3^
*Z* = 4
Mo *K*α radiation
μ = 0.61 mm^−1^
*T* = 100 (2) K
0.32 × 0.08 × 0.07 mm

#### Data collection

Bruker APEXII CCD area-detector’ diffractometer
Absorption correction: multi-scan (*SADABS*; Bruker, 2005 ▶) *T*_min_ = 0.829, *T*_max_ = 0.959
24511 measured reflections
5229 independent reflections
3694 reflections with *I* > 2σ(*I*)
*R*_int_ = 0.078

#### Refinement

*R*[*F* ^2^ > 2σ(*F* ^2^)] = 0.042
*wR*(*F* ^2^) = 0.104
*S* = 1.02
5229 reflections
336 parameters
H-atom parameters constrained
Δρ_max_ = 0.47 e Å^−3^
Δρ_min_ = −0.57 e Å^−3^

### 

Data collection: *APEX2* (Bruker, 2005 ▶); cell refinement: *SAINT* (Bruker, 2005 ▶); data reduction: *SAINT*; program(s) used to solve structure: *SHELXS97* (Sheldrick, 2008 ▶); program(s) used to refine structure: *SHELXL97* (Sheldrick, 2008 ▶); molecular graphics: *SHELXTL* (Sheldrick, 2008 ▶); software used to prepare material for publication: *SHELXTL*.

## Supplementary Material

Crystal structure: contains datablocks I, global. DOI: 10.1107/S1600536808027347/ym2072sup1.cif

Structure factors: contains datablocks I. DOI: 10.1107/S1600536808027347/ym2072Isup2.hkl

Additional supplementary materials:  crystallographic information; 3D view; checkCIF report

## Figures and Tables

**Table 1:** Hydrogen-bond geometry (Å, °)

*D*—H⋯*A*	*D*—H	H⋯*A*	*D*⋯*A*	*D*—H⋯*A*
N3—H3*NA*⋯O2^i^	0.87	2.03	2.840 (3)	154
N4—H4*NB*⋯O11^i^	0.87	2.09	2.958 (3)	173
N4—H4*NA*⋯O3^ii^	0.87	2.33	3.122 (3)	151
N5—H5*NB*⋯O8	0.87	1.99	2.831 (3)	162
N5—H5*NA*⋯O2^i^	0.87	2.01	2.812 (3)	152
O5—H5*O*⋯O12	0.80	1.77	2.560 (2)	169
O10—H10*O*⋯O11^iii^	0.80	1.83	2.624 (3)	173
O11—H11*C*⋯O1	0.82	1.92	2.719 (2)	163
O11—H11*D*⋯O7^iv^	0.82	1.90	2.709 (3)	169
O12—H12*A*⋯O9^v^	0.82	2.01	2.809 (3)	165
O12—H12*B*⋯O4^ii^	0.82	2.05	2.872 (3)	177
C16—H16*A*⋯O3^ii^	0.95	2.49	3.265 (3)	139
C18—H18*A*⋯O12^vi^	0.95	2.59	3.279 (3)	130

Symmetry codes: (i) 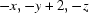; (ii) 
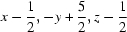
; (iii) 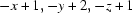; (iv) 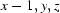; (v) 
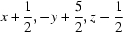
; (vi) 
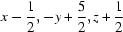
.

**Fig. 1.**
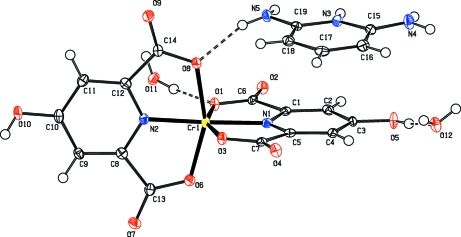
Molecular structure of (pydaH)[Cr(hypydc)2].2H2O, hydrogen bonds are shown as dashed lines and thermal ellipsoids are at 50% probability level.

**Fig. 2.**
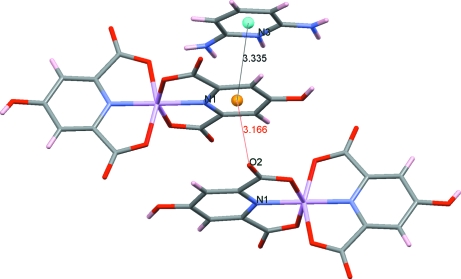
The π–πand C—O···π stacking interactions with the distances.

**Fig. 3.**
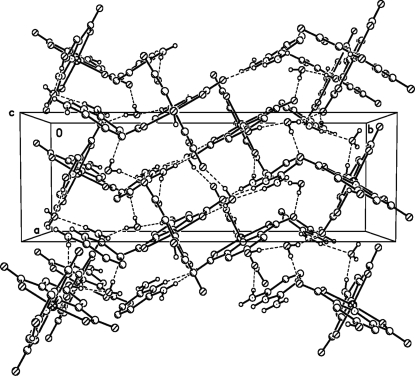
Crystal packing of the title compound showing the three dimensional H-bonded framework.

## supplementary crystallographic information

### Comment

The molecular structure of the title compound is shown in Fig. 1. Hydrogen bond geometries are given in Table 1. According to the crystal structure, the title compound is composed of an anionic complex, [Cr(hypydc)_2_]^-^, protonated diamino-2,6-pyridinium as a counter ion, (pydaH)^+^, and two uncoordinated water molecules. This compound crystallizes in the monoclinic system, space group *P*2_1_/*n* with four molecules in the unit cell.

The Cr^III^ atom is six-coordinated by two 4-hydroxypyridine-2,6-dicarboxylate, (hypydc)^2-^, groups which act as tridentate ligand through one pyridine N atom and two carboxylate O atoms. N1 and N2 atoms of two (hypydc)^2-^ fragments occupy the axial position, while O1, O3, O6 and O8 atoms form the equatorial plane. The N1—Cr1—N2 angle [177.33 (9)°] deviates slightly from linearity. Therefore, the coordination geometry around Cr^III ^can be described as distorted octahedral. The bond angles O6—Cr1—O1, O3—Cr1—O6, O3—Cr1—O8 and O1—Cr1—O8 (93.28 (7)°, 93.82 (7)°, 90.88 (7)° and 91.46 (7)°, respectively), and the O1—Cr1—O6—C13 and O8—Cr1—O1—C6 torsion angles (-99.81 (17)° and (97.77 (17)°, respectively) show that these two (hypydc)^2-^ groups are almost perpendicular. Furthermore, the bond angles O1—Cr1—O3 [156.06 (7)°] and O6—Cr1—O8 [156.99 (7)°] indicate that four carboxylate groups of the two dianions are in a flattened tetrahedral arrangement around the Cr^III^ atom.

In the crystal structure of (pydaH)[Cr(hypydc)_2_].2H_2_O, the spaces between to layers of [Cr(hypydc)_2_]^-^ anions are filled with (pydaH)^+ ^cations and water molecules. There are also *π*-*π*stacking interactions between the aromatic ring of the coordinated (hypydc)^2– ^anion and the aromatic ring of the (pydaH)^+^ cation with distances of 3.3353 (14) Å for *Cg*1···*Cg*2. There are also C—O···*π* stacking interactions between C6–O2 and *Cg*1 with O···*π* distance of 3.166 (2) Å (1 - *x*, 2 - *y*, -*z*) [*Cg*1 and *Cg*2 are the centroid for (N1/C1—C5) and (N3/C15—C19) rings, respectively] (Fig. 2).

Intermolecular O—H···O, N—H···O and C—H···O hydrogen bonding with D···A ranging from 2.560 (2) to 3.279 (3) Å, ionpairing, *π*–*π*and C—O···*π*stacking interactions seem to be effective in the stabilization of the crystal structure, resulting in the formation of an interesting supramolecular structure (Fig. 3).

### Experimental

An aqueous solution of CrCl_3 _(24 mg, 0.15 mmol), pyridine-2,6-diamine (32 mg, 0.3 mmol) and 4-Hydroxypyridine-2,6-dicarboxylic acid (54 mg, 0.3 mmol) was added to each other in a 1:2:2 molar ratio, and the reaction mixture was heated at about 50°C for 4 h. Violet crystals of the title compound were obtained from the solution after two weeks at room temperature.

### Refinement

The hydrogen atoms of NH2 and OH groups and also H atoms of water molecule were found in difference Fourier synthesis. The hydrogen atoms of the H(C) atom positions were calculated. All Hydrogen atoms were refined in isotropic approximation in riding model with the *U*_iso_(H) parameters equal to 1.2 *U*_eq_(Xi), where U(Xi) the equivalent thermal parameters of the carbon or nitrogen or oxygen atom to which corresponding H atom is bonded.

### Crystal data

**Table:**

(C_5_H_8_N_3_)[Cr(C_7_H_3_N_1_O_5_)_2_]·2H_2_O	*F*_000_ = 1148
*M**_r_* = 560.38	*D*_x_ = 1.718 Mg m^−^^3^
Monoclinic, *P*2_1_/*n*	Mo *K*α radiation λ = 0.71073 Å
Hall symbol: -P 2yn	Cell parameters from 1320 reflections
*a* = 6.9590 (4) Å	θ = 3–28º
*b* = 20.9904 (12) Å	µ = 0.61 mm^−^^1^
*c* = 14.8951 (8) Å	*T* = 100 (2) K
β = 95.3030 (13)º	Needle, black
*V* = 2166.4 (2) Å^3^	0.32 × 0.08 × 0.07 mm
*Z* = 4	

### Data collection

**Table:**

Bruker APEXII CCD area-detector' diffractometer	5229 independent reflections
Radiation source: fine-focus sealed tube	3694 reflections with *I* > 2σ(*I*)
Monochromator: graphite	*R*_int_ = 0.078
*T* = 100(2) K	θ_max_ = 28.0º
ω scans	θ_min_ = 1.7º
Absorption correction: multi-scan(SADABS; Bruker, 2005)	*h* = −9→9
*T*_min_ = 0.829, *T*_max_ = 0.959	*k* = −27→27
24511 measured reflections	*l* = −19→19

### Refinement

**Table:**

Refinement on *F*^2^	Secondary atom site location: difference Fourier map
Least-squares matrix: full	Hydrogen site location: mixed
*R*[*F*^2^ > 2σ(*F*^2^)] = 0.042	H-atom parameters constrained
*wR*(*F*^2^) = 0.104	*w* = 1/[σ^2^(*F*_o_^2^) + (0.0468*P*)^2^ + 0.9675*P*] where *P* = (*F*_o_^2^ + 2*F*_c_^2^)/3
*S* = 1.02	(Δ/σ)_max_ = 0.002
5229 reflections	Δρ_max_ = 0.47 e Å^−^^3^
336 parameters	Δρ_min_ = −0.57 e Å^−^^3^
Primary atom site location: structure-invariant direct methods	Extinction correction: none

### Special details

**Table:**

Geometry. All e.s.d.'s (except the e.s.d. in the dihedral angle between two l.s. planes) are estimated using the full covariance matrix. The cell e.s.d.'s are taken into account individually in the estimation of e.s.d.'s in distances, angles and torsion angles; correlations between e.s.d.'s in cell parameters are only used when they are defined by crystal symmetry. An approximate (isotropic) treatment of cell e.s.d.'s is used for estimating e.s.d.'s involving l.s. planes.
Refinement. Refinement of *F*^2^ against ALL reflections. The weighted *R*-factor *wR* and goodness of fit *S* are based on *F*^2^, conventional *R*-factors *R* are based on *F*, with *F* set to zero for negative *F*^2^. The threshold expression of *F*^2^ > σ(*F*^2^) is used only for calculating *R*-factors(gt) *etc*. and is not relevant to the choice of reflections for refinement. *R*-factors based on *F*^2^ are statistically about twice as large as those based on *F*, and *R*- factors based on ALL data will be even larger.

### Fractional atomic coordinates and isotropic or equivalent isotropic displacement parameters (Å^2^)

**Table:**

	*x*	*y*	*z*	*U*_iso_*/*U*_eq_	
Cr1	0.46263 (6)	1.098248 (19)	0.20578 (3)	0.00992 (10)	
O1	0.3226 (2)	1.01686 (8)	0.17083 (11)	0.0124 (4)	
O2	0.1871 (3)	0.95887 (8)	0.05618 (11)	0.0141 (4)	
O3	0.5836 (2)	1.18207 (8)	0.18501 (11)	0.0127 (4)	
O4	0.6584 (3)	1.25241 (8)	0.08081 (12)	0.0154 (4)	
O5	0.3766 (3)	1.11140 (8)	−0.20081 (11)	0.0166 (4)	
H5O	0.4037	1.1423	−0.2284	0.020*	
O6	0.7149 (2)	1.05418 (8)	0.23449 (11)	0.0138 (4)	
O7	0.9177 (3)	1.01550 (9)	0.34882 (12)	0.0192 (4)	
O8	0.2157 (2)	1.14101 (8)	0.23077 (11)	0.0135 (4)	
O9	0.0389 (3)	1.17639 (9)	0.33928 (12)	0.0174 (4)	
O10	0.5003 (3)	1.09070 (9)	0.61131 (11)	0.0190 (4)	
H10O	0.6029	1.0846	0.6388	0.023*	
O11	0.1788 (3)	0.93417 (8)	0.28815 (12)	0.0159 (4)	
H11D	0.0901	0.9544	0.3071	0.019*	
H11C	0.2300	0.9525	0.2480	0.046 (11)*	
O12	0.4442 (3)	1.21870 (8)	−0.27326 (12)	0.0170 (4)	
H12A	0.4497	1.2508	−0.2418	0.020*	
H12B	0.3656	1.2273	−0.3160	0.020*	
N1	0.4410 (3)	1.10250 (10)	0.07265 (13)	0.0106 (4)	
N2	0.4719 (3)	1.09238 (9)	0.33828 (13)	0.0105 (4)	
N3	−0.0320 (3)	1.16527 (10)	−0.07279 (13)	0.0117 (4)	
H3NA	−0.0906	1.1294	−0.0859	0.014*	
N4	−0.0433 (3)	1.19284 (11)	−0.22368 (14)	0.0178 (5)	
H4NB	−0.0924	1.1563	−0.2413	0.021*	
H4NA	−0.0025	1.2175	−0.2648	0.032 (9)*	
N5	−0.0454 (3)	1.13054 (10)	0.07407 (14)	0.0135 (4)	
H5NB	0.0119	1.1350	0.1281	0.016*	
H5NA	−0.0986	1.0961	0.0506	0.016*	
C1	0.3476 (3)	1.05598 (11)	0.02427 (16)	0.0101 (5)	
C2	0.3233 (4)	1.05809 (12)	−0.06822 (16)	0.0117 (5)	
H2A	0.2573	1.0250	−0.1019	0.014*	
C3	0.3993 (3)	1.11092 (11)	−0.11176 (16)	0.0108 (5)	
C4	0.4932 (3)	1.15980 (12)	−0.05965 (16)	0.0110 (5)	
H4A	0.5443	1.1959	−0.0876	0.013*	
C5	0.5088 (3)	1.15376 (11)	0.03232 (16)	0.0108 (5)	
C6	0.2781 (3)	1.00555 (11)	0.08605 (16)	0.0105 (5)	
C7	0.5931 (3)	1.20100 (12)	0.10225 (16)	0.0111 (5)	
C8	0.6272 (4)	1.06604 (12)	0.38294 (16)	0.0123 (5)	
C9	0.6455 (4)	1.06333 (12)	0.47589 (16)	0.0122 (5)	
H9A	0.7555	1.0444	0.5078	0.015*	
C10	0.4959 (4)	1.08960 (11)	0.52222 (16)	0.0137 (5)	
C11	0.3335 (4)	1.11654 (12)	0.47336 (17)	0.0137 (5)	
H11A	0.2305	1.1339	0.5032	0.016*	
C12	0.3287 (4)	1.11698 (11)	0.38087 (16)	0.0117 (5)	
C13	0.7689 (4)	1.04231 (12)	0.31917 (16)	0.0128 (5)	
C14	0.1782 (4)	1.14746 (11)	0.31450 (16)	0.0118 (5)	
C15	0.0192 (4)	1.20629 (12)	−0.13776 (17)	0.0132 (5)	
C16	0.1308 (4)	1.25899 (12)	−0.11129 (17)	0.0149 (5)	
H16A	0.1713	1.2879	−0.1549	0.018*	
C17	0.1819 (4)	1.26871 (12)	−0.02048 (17)	0.0138 (5)	
H17A	0.2600	1.3044	−0.0023	0.017*	
C18	0.1230 (4)	1.22815 (12)	0.04490 (17)	0.0135 (5)	
H18A	0.1551	1.2368	0.1071	0.016*	
C19	0.0162 (4)	1.17463 (12)	0.01759 (16)	0.0122 (5)	

### Atomic displacement parameters (Å^2^)

**Table:**

	*U*^11^	*U*^22^	*U*^33^	*U*^12^	*U*^13^	*U*^23^
Cr1	0.0125 (2)	0.0104 (2)	0.00680 (19)	0.00029 (16)	0.00056 (14)	−0.00067 (15)
O1	0.0170 (9)	0.0115 (9)	0.0083 (9)	−0.0017 (7)	0.0000 (7)	0.0004 (6)
O2	0.0167 (9)	0.0123 (9)	0.0131 (9)	−0.0027 (7)	0.0006 (7)	−0.0010 (7)
O3	0.0161 (9)	0.0135 (9)	0.0084 (9)	−0.0023 (7)	0.0003 (7)	−0.0013 (7)
O4	0.0189 (10)	0.0120 (9)	0.0149 (10)	−0.0027 (8)	−0.0011 (7)	0.0010 (7)
O5	0.0284 (11)	0.0143 (10)	0.0068 (9)	−0.0023 (8)	0.0011 (7)	0.0021 (7)
O6	0.0143 (9)	0.0156 (9)	0.0114 (9)	0.0019 (7)	0.0014 (7)	−0.0015 (7)
O7	0.0170 (10)	0.0241 (11)	0.0161 (10)	0.0082 (8)	−0.0007 (7)	−0.0006 (8)
O8	0.0135 (9)	0.0175 (9)	0.0092 (9)	0.0023 (7)	−0.0002 (7)	−0.0007 (7)
O9	0.0177 (10)	0.0214 (10)	0.0134 (10)	0.0071 (8)	0.0029 (7)	−0.0001 (7)
O10	0.0214 (10)	0.0279 (11)	0.0073 (9)	0.0029 (8)	−0.0010 (7)	−0.0002 (7)
O11	0.0169 (10)	0.0181 (10)	0.0136 (9)	0.0020 (8)	0.0052 (7)	0.0022 (7)
O12	0.0230 (10)	0.0145 (9)	0.0128 (9)	−0.0013 (8)	−0.0018 (7)	0.0020 (7)
N1	0.0102 (10)	0.0101 (10)	0.0117 (10)	0.0019 (8)	0.0014 (8)	−0.0019 (8)
N2	0.0125 (10)	0.0100 (10)	0.0088 (10)	0.0002 (8)	0.0003 (8)	−0.0009 (8)
N3	0.0133 (11)	0.0102 (10)	0.0118 (11)	−0.0008 (8)	0.0018 (8)	0.0010 (8)
N4	0.0215 (12)	0.0190 (12)	0.0125 (11)	−0.0055 (10)	−0.0010 (9)	0.0041 (9)
N5	0.0147 (11)	0.0158 (11)	0.0097 (10)	−0.0018 (9)	−0.0007 (8)	−0.0002 (8)
C1	0.0092 (12)	0.0094 (12)	0.0118 (12)	0.0024 (9)	0.0024 (9)	−0.0006 (9)
C2	0.0116 (12)	0.0098 (12)	0.0132 (13)	0.0025 (9)	−0.0008 (9)	−0.0012 (9)
C3	0.0117 (12)	0.0140 (13)	0.0066 (11)	0.0042 (10)	0.0001 (9)	−0.0002 (9)
C4	0.0102 (12)	0.0115 (12)	0.0114 (12)	0.0030 (9)	0.0018 (9)	0.0018 (9)
C5	0.0081 (12)	0.0110 (12)	0.0134 (13)	0.0015 (9)	0.0017 (9)	−0.0007 (9)
C6	0.0103 (12)	0.0116 (12)	0.0099 (12)	0.0025 (10)	0.0030 (9)	0.0002 (9)
C7	0.0088 (12)	0.0133 (12)	0.0113 (12)	0.0031 (10)	0.0011 (9)	−0.0018 (9)
C8	0.0129 (13)	0.0105 (12)	0.0134 (13)	−0.0018 (10)	−0.0002 (10)	−0.0004 (9)
C9	0.0140 (13)	0.0112 (12)	0.0110 (12)	0.0022 (10)	−0.0014 (9)	0.0005 (9)
C10	0.0201 (13)	0.0110 (12)	0.0098 (12)	−0.0014 (10)	0.0008 (10)	0.0015 (9)
C11	0.0160 (13)	0.0123 (12)	0.0132 (13)	−0.0016 (10)	0.0037 (10)	−0.0013 (9)
C12	0.0122 (12)	0.0100 (12)	0.0124 (12)	−0.0020 (9)	−0.0011 (9)	−0.0021 (9)
C13	0.0154 (13)	0.0117 (12)	0.0113 (12)	−0.0009 (10)	0.0011 (10)	−0.0017 (9)
C14	0.0145 (13)	0.0109 (12)	0.0098 (12)	−0.0008 (10)	0.0005 (9)	0.0003 (9)
C15	0.0130 (13)	0.0143 (13)	0.0123 (13)	0.0050 (10)	0.0016 (10)	0.0030 (9)
C16	0.0150 (13)	0.0125 (13)	0.0173 (14)	0.0019 (10)	0.0030 (10)	0.0039 (10)
C17	0.0111 (12)	0.0088 (12)	0.0214 (14)	0.0015 (10)	0.0010 (10)	−0.0017 (10)
C18	0.0120 (13)	0.0130 (13)	0.0154 (13)	0.0014 (10)	0.0000 (10)	−0.0025 (10)
C19	0.0090 (12)	0.0152 (13)	0.0123 (13)	0.0034 (10)	−0.0001 (9)	−0.0002 (9)

### Geometric parameters (Å, °)

**Table:**

Cr1—N2	1.973 (2)	N4—C15	1.343 (3)
Cr1—N1	1.977 (2)	N4—H4NB	0.8700
Cr1—O3	1.9872 (17)	N4—H4NA	0.8700
Cr1—O6	1.9957 (18)	N5—C19	1.347 (3)
Cr1—O8	2.0036 (17)	N5—H5NB	0.8700
Cr1—O1	2.0111 (17)	N5—H5NA	0.8701
O1—C6	1.294 (3)	C1—C2	1.373 (3)
O2—C6	1.227 (3)	C1—C6	1.511 (3)
O3—C7	1.303 (3)	C2—C3	1.412 (3)
O4—C7	1.225 (3)	C2—H2A	0.9500
O5—C3	1.321 (3)	C3—C4	1.410 (3)
O5—H5O	0.7999	C4—C5	1.370 (3)
O6—C13	1.306 (3)	C4—H4A	0.9500
O7—C13	1.225 (3)	C5—C7	1.516 (3)
O8—C14	1.305 (3)	C8—C9	1.380 (3)
O9—C14	1.229 (3)	C8—C13	1.515 (3)
O10—C10	1.325 (3)	C9—C10	1.413 (3)
O10—H10O	0.8000	C9—H9A	0.9500
O11—H11D	0.8199	C10—C11	1.405 (4)
O11—H11C	0.8201	C11—C12	1.375 (3)
O12—H12A	0.8198	C11—H11A	0.9500
O12—H12B	0.8201	C12—C14	1.514 (3)
N1—C5	1.339 (3)	C15—C16	1.388 (4)
N1—C1	1.345 (3)	C16—C17	1.382 (4)
N2—C12	1.334 (3)	C16—H16A	0.9500
N2—C8	1.335 (3)	C17—C18	1.384 (4)
N3—C15	1.367 (3)	C17—H17A	0.9500
N3—C19	1.371 (3)	C18—C19	1.387 (3)
N3—H3NA	0.8701	C18—H18A	0.9500
			
N2—Cr1—N1	177.33 (9)	C5—C4—H4A	120.9
N2—Cr1—O3	103.64 (8)	C3—C4—H4A	120.9
N1—Cr1—O3	78.35 (8)	N1—C5—C4	121.6 (2)
N2—Cr1—O6	79.04 (8)	N1—C5—C7	110.3 (2)
N1—Cr1—O6	102.70 (8)	C4—C5—C7	128.0 (2)
O3—Cr1—O6	93.82 (7)	O2—C6—O1	124.7 (2)
N2—Cr1—O8	77.96 (8)	O2—C6—C1	121.4 (2)
N1—Cr1—O8	100.31 (8)	O1—C6—C1	113.9 (2)
O3—Cr1—O8	90.88 (7)	O4—C7—O3	124.6 (2)
O6—Cr1—O8	156.99 (7)	O4—C7—C5	121.8 (2)
N2—Cr1—O1	100.15 (8)	O3—C7—C5	113.6 (2)
N1—Cr1—O1	77.79 (8)	N2—C8—C9	120.8 (2)
O3—Cr1—O1	156.06 (7)	N2—C8—C13	111.6 (2)
O6—Cr1—O1	93.28 (7)	C9—C8—C13	127.6 (2)
O8—Cr1—O1	91.46 (7)	C8—C9—C10	118.0 (2)
C6—O1—Cr1	118.37 (15)	C8—C9—H9A	121.0
C7—O3—Cr1	118.46 (15)	C10—C9—H9A	121.0
C3—O5—H5O	120.7	O10—C10—C11	117.1 (2)
C13—O6—Cr1	117.65 (15)	O10—C10—C9	123.1 (2)
C14—O8—Cr1	118.36 (15)	C11—C10—C9	119.8 (2)
C10—O10—H10O	116.5	C12—C11—C10	117.7 (2)
H11D—O11—H11C	113.6	C12—C11—H11A	121.1
H12A—O12—H12B	104.7	C10—C11—H11A	121.1
C5—N1—C1	121.1 (2)	N2—C12—C11	121.6 (2)
C5—N1—Cr1	119.27 (16)	N2—C12—C14	110.8 (2)
C1—N1—Cr1	119.54 (16)	C11—C12—C14	127.5 (2)
C12—N2—C8	121.9 (2)	O7—C13—O6	126.4 (2)
C12—N2—Cr1	119.67 (17)	O7—C13—C8	120.2 (2)
C8—N2—Cr1	118.33 (16)	O6—C13—C8	113.4 (2)
C15—N3—C19	123.3 (2)	O9—C14—O8	124.9 (2)
C15—N3—H3NA	122.3	O9—C14—C12	122.0 (2)
C19—N3—H3NA	114.3	O8—C14—C12	113.1 (2)
C15—N4—H4NB	123.8	N4—C15—N3	117.4 (2)
C15—N4—H4NA	116.6	N4—C15—C16	124.2 (2)
H4NB—N4—H4NA	117.1	N3—C15—C16	118.5 (2)
C19—N5—H5NB	111.0	C17—C16—C15	118.9 (2)
C19—N5—H5NA	117.8	C17—C16—H16A	120.6
H5NB—N5—H5NA	127.1	C15—C16—H16A	120.6
N1—C1—C2	121.5 (2)	C16—C17—C18	122.1 (2)
N1—C1—C6	110.4 (2)	C16—C17—H17A	118.9
C2—C1—C6	128.1 (2)	C18—C17—H17A	118.9
C1—C2—C3	118.0 (2)	C17—C18—C19	118.5 (2)
C1—C2—H2A	121.0	C17—C18—H18A	120.7
C3—C2—H2A	121.0	C19—C18—H18A	120.7
O5—C3—C4	123.6 (2)	N5—C19—N3	116.8 (2)
O5—C3—C2	116.9 (2)	N5—C19—C18	124.5 (2)
C4—C3—C2	119.5 (2)	N3—C19—C18	118.7 (2)
C5—C4—C3	118.2 (2)		
			
N2—Cr1—O1—C6	175.80 (17)	C3—C4—C5—C7	−176.5 (2)
N1—Cr1—O1—C6	−2.46 (16)	Cr1—O1—C6—O2	−177.36 (19)
O3—Cr1—O1—C6	2.3 (3)	Cr1—O1—C6—C1	2.3 (3)
O6—Cr1—O1—C6	−104.75 (17)	N1—C1—C6—O2	179.2 (2)
O8—Cr1—O1—C6	97.77 (17)	C2—C1—C6—O2	−0.1 (4)
N2—Cr1—O3—C7	−177.73 (16)	N1—C1—C6—O1	−0.5 (3)
N1—Cr1—O3—C7	0.44 (17)	C2—C1—C6—O1	−179.8 (2)
O6—Cr1—O3—C7	102.64 (17)	Cr1—O3—C7—O4	177.52 (19)
O8—Cr1—O3—C7	−99.91 (17)	Cr1—O3—C7—C5	−0.6 (3)
O1—Cr1—O3—C7	−4.3 (3)	N1—C5—C7—O4	−177.7 (2)
N2—Cr1—O6—C13	−0.10 (17)	C4—C5—C7—O4	0.4 (4)
N1—Cr1—O6—C13	−178.02 (17)	N1—C5—C7—O3	0.5 (3)
O3—Cr1—O6—C13	103.07 (17)	C4—C5—C7—O3	178.6 (2)
O8—Cr1—O6—C13	1.7 (3)	C12—N2—C8—C9	−0.1 (4)
O1—Cr1—O6—C13	−99.81 (17)	Cr1—N2—C8—C9	176.98 (18)
N2—Cr1—O8—C14	2.31 (17)	C12—N2—C8—C13	−179.5 (2)
N1—Cr1—O8—C14	−179.77 (17)	Cr1—N2—C8—C13	−2.4 (3)
O3—Cr1—O8—C14	−101.46 (17)	N2—C8—C9—C10	−0.4 (4)
O6—Cr1—O8—C14	0.5 (3)	C13—C8—C9—C10	178.9 (2)
O1—Cr1—O8—C14	102.37 (17)	C8—C9—C10—O10	−178.7 (2)
O3—Cr1—N1—C5	−0.11 (17)	C8—C9—C10—C11	0.9 (4)
O6—Cr1—N1—C5	−91.45 (18)	O10—C10—C11—C12	178.6 (2)
O8—Cr1—N1—C5	88.65 (18)	C9—C10—C11—C12	−1.0 (4)
O1—Cr1—N1—C5	177.91 (19)	C8—N2—C12—C11	0.0 (4)
O3—Cr1—N1—C1	−175.84 (19)	Cr1—N2—C12—C11	−176.99 (18)
O6—Cr1—N1—C1	92.83 (18)	C8—N2—C12—C14	176.2 (2)
O8—Cr1—N1—C1	−87.08 (18)	Cr1—N2—C12—C14	−0.8 (3)
O1—Cr1—N1—C1	2.18 (17)	C10—C11—C12—N2	0.5 (4)
O3—Cr1—N2—C12	87.31 (19)	C10—C11—C12—C14	−175.0 (2)
O6—Cr1—N2—C12	178.62 (19)	Cr1—O6—C13—O7	179.5 (2)
O8—Cr1—N2—C12	−0.64 (18)	Cr1—O6—C13—C8	−1.1 (3)
O1—Cr1—N2—C12	−89.97 (19)	N2—C8—C13—O7	−178.4 (2)
O3—Cr1—N2—C8	−89.81 (18)	C9—C8—C13—O7	2.3 (4)
O6—Cr1—N2—C8	1.50 (18)	N2—C8—C13—O6	2.2 (3)
O8—Cr1—N2—C8	−177.77 (19)	C9—C8—C13—O6	−177.1 (2)
O1—Cr1—N2—C8	92.91 (18)	Cr1—O8—C14—O9	174.9 (2)
C5—N1—C1—C2	2.1 (3)	Cr1—O8—C14—C12	−3.3 (3)
Cr1—N1—C1—C2	177.73 (18)	N2—C12—C14—O9	−175.7 (2)
C5—N1—C1—C6	−177.3 (2)	C11—C12—C14—O9	0.2 (4)
Cr1—N1—C1—C6	−1.6 (3)	N2—C12—C14—O8	2.6 (3)
N1—C1—C2—C3	−0.1 (3)	C11—C12—C14—O8	178.5 (2)
C6—C1—C2—C3	179.1 (2)	C19—N3—C15—N4	−177.6 (2)
C1—C2—C3—O5	179.0 (2)	C19—N3—C15—C16	2.1 (4)
C1—C2—C3—C4	−1.2 (3)	N4—C15—C16—C17	178.2 (2)
O5—C3—C4—C5	−179.6 (2)	N3—C15—C16—C17	−1.5 (4)
C2—C3—C4—C5	0.6 (3)	C15—C16—C17—C18	−1.0 (4)
C1—N1—C5—C4	−2.7 (3)	C16—C17—C18—C19	2.8 (4)
Cr1—N1—C5—C4	−178.40 (18)	C15—N3—C19—N5	179.4 (2)
C1—N1—C5—C7	175.5 (2)	C15—N3—C19—C18	−0.3 (4)
Cr1—N1—C5—C7	−0.2 (3)	C17—C18—C19—N5	178.2 (2)
C3—C4—C5—N1	1.4 (4)	C17—C18—C19—N3	−2.1 (4)

### Hydrogen-bond geometry (Å, °)

**Table:**

*D*—H···*A*	*D*—H	H···*A*	*D*···*A*	*D*—H···*A*
N3—H3NA···O2^i^	0.87	2.03	2.840 (3)	154
N4—H4NB···O11^i^	0.87	2.09	2.958 (3)	173
N4—H4NA···O3^ii^	0.87	2.33	3.122 (3)	151
N5—H5NB···O8	0.87	1.99	2.831 (3)	162
N5—H5NA···O2^i^	0.87	2.01	2.812 (3)	152
O5—H5O···O12	0.80	1.77	2.560 (2)	169
O10—H10O···O11^iii^	0.80	1.83	2.624 (3)	173
O11—H11C···O1	0.82	1.92	2.719 (2)	163
O11—H11D···O7^iv^	0.82	1.90	2.709 (3)	169
O12—H12A···O9^v^	0.82	2.01	2.809 (3)	165
O12—H12B···O4^ii^	0.82	2.05	2.872 (3)	177
C16—H16A···O3^ii^	0.95	2.49	3.265 (3)	139
C18—H18A···O12^vi^	0.95	2.59	3.279 (3)	130

Symmetry codes: (i) −*x*, −*y*+2, −*z*; (ii) *x*−1/2, −*y*+5/2, *z*−1/2;  (iii) −*x*+1, −*y*+2, −*z*+1; (iv) *x*−1, *y*, *z*; (v) *x*+1/2, −*y*+5/2, *z*−1/2; (vi) *x*−1/2, −*y*+5/2, *z*+1/2.

## References

[bb1] Aghabozorg, H., Ghadermazi, M., Soleimannejad, J. & Sheshmani, S. (2007). *Acta Cryst.* E**63**, m1917–m1918.

[bb2] Aghabozorg, H., Manteghi, F. & Sheshmani, S. (2008). *J. Iran. Chem. Soc. ***5**, 184–227.

[bb3] Aghabozorg, H., Saadaty, S., Motyeian, E., Ghadermazi, M. & Manteghi, F. (2008). *Acta Cryst* E**64**, m466–m467. 10.1107/S1600536808003930 PMC2960790 21201857

[bb4] Bruker (2005). *APEX2*, *SAINT and *SADABS** Bruker AXS Inc., Madison, Wisconsin, USA.

[bb5] Ranjbar, M., Aghabozorg, H., Moghimi, A. & Yanovsky, A. (2001). *Z. Kristallogr* *New Cryst. Struct* **216**, 626–628.

[bb6] Sheldrick, G. M. (2008). *Acta Cryst.* A**64**, 112–122. 10.1107/S0108767307043930 18156677

[bb7] Soleimannejad, J., Aghabozorg, H. & Hooshmand, S. (2008). *Acta Cryst.* E**64**, m564–m565. 10.1107/S1600536808006594 PMC2960993 21202019

